# On the Use of Chloroform in Europe

**Published:** 1853-01

**Authors:** 


*Chloroform.*—This furnished a topic of much interest in Edinburgh. At breakfast, one morning, Prof. Simpson met me with these tidings from America. "Prof. C., the Boston Journal says that three more cases of death from chloroform have recently occurred in your country." I now asked again if he had met with any untoward results from its use in his own practice—with any thing which had produced in his mind the least doubt as to its entire safety. He said, no—he had not met with any accident from the use of chloroform. I knew Prof. S. introduced chloroform into the practice of midwifery, and of surgery, and that it might be said that such a relation to it would bias his opinions concerning it. I therefore looked for its use elsewhere, in the hands of others. The same answer came. Here were patients undergoing surgical operations; and choloform, in its fullest use, in these, was found to be as safe as elsewhere. It was my privilege to see it used in midwifery, and it was as manageable as any other medicinal agent of real power, in any employment of it. There was one application of it, which seemed to me as much of a test trial as well could be. This was its employment to produce insensibility in painful diseases, and as a means of diagnosis. I say a test or trial use, and for this reason. In the surgical cases which have been fatal under the use of chloroform, it has been employed to produce insensibility as a *preparation* for operations. It has not been when employed in the *midst of operations*, and to *continue* insensibility during the progress of the knife, that it has been fatal, but when used as a *preparation*, and especially in cases of slight surgical interest. It has never been fatal in midwifery, and the explanation is, that in these long-continued cases of suffering such a condition of nervous power is produced as to modify the action of chloroform. But in its use for diagnosis, such condition has probably been but partially produced, and still, in these, chloroform was perfectly safe. It was suggested that the occasional mortality after the use of chloroform may be owing to its impurity. I called, with Prof. Simpson, on Messrs. Duncan, Flockhart & Co., and examined their chloroform, and compared it with other specimens obtained from other chemists and druggists. The process was a very simple one, and though it showed a difference among kinds, it did not teach on what the difference depended. The process consisted in dropping a little chloroform on the back of the hand, and when the part was dry it was smelled to. No smell remained after the trial with Duncan & Co.'s. A disagreeable odor remained after another specimen was tested in the same way. Now though some impurity may exist in a specimen of chloroform, the question is not settled how far such would affect its medicinal use. I was assured that Duncan's chloroform was in extensive use in Edinburgh, and that no untoward results had marked its employment. Nor had such result, as far as I heard, followed the use of what seemed less pure chloroform. Messrs. Duncan & Co. told me that their weekly orders from London was between one and two hundred pounds of chloroform. Much more must be supplied by other manufacturers, for, as far as learned, this is the principal anæsthetic employed in Great Britain and on the Continent. Supposing impurity to have had no agency in producing the deaths which have followed the use of chloroform, we look to predisposition either of idiosyncrasy, or other, if such exist, and which may involve the same fatal results. The question which arises here is quite as difficult to answer as that which impurity of the article may suggest. In other words we may know as little of one as the other, and the concurrence of both may be as little understood. We must then take the facts as they are, and each decide for himself what his course concerning anæsthetics shall be. I have brought with me a specimen of Edinburgh chloroform. One of our best druggists has examined this, and expressed himself so entirely pleased with it that he means to order one hundred pounds of it from the house of Duncan & Co., in Edinburgh.

It is not my purpose to discuss the question of the comparative advantages and dangers of chloroform and sulphuric ether. I gathered abroad that chloroform is much employed. In England, it has been seriously and strongly condemned by some, while many use it. In Scotland, I did not hear the word *ether* uttered in this connection. With us there is a divided opinion against it. One or two employ chloric ether—a solution of ether in alcohol—a tincture of chloroform, the produce of distillation, and of simple mixture with alcohol, being precisely the same.

Others say that this has its sole power in the chloroform, as the brandy and water dram has its main claim in the brandy; and we have one case narrated, and which occurred here, in which death took place almost immediately after the inhalation of chloric ether. Many here use chloroform altogether, and without any more distrust of its perfectly safe powers than the Faculty in Edinburgh have. By far the greater number use sulphuric ether, and are entirely satisfied with it. It is effectual, say they, and safe. For myself, I mostly use ether. There are cases in which this fails; and it may be when an important operation in midwifery is to be done. In such, I substitute chloroform, and have never had cause to regret its employment. Since I returned from abroad, I have had consultations in difficult labor, and in one half, I have found the attendant using chloroform—in the other, ether; the results were alike excellent in both. The forceps was used in these, in a state of perfect unconsciousness, and both mothers and children have done alike well. There is not on record a case of death after chloroform in midwifery practice, and I have no reason to believe that unrecorded cases have happened. In surgery, deaths have occurred. In these, death has followed the use of chloroform as a *preparation* for the operation. In midwifery, and medicine, its use is very much confined to advanced periods of the labor, or of the disease. In one complication of labor—convulsions—chloroform has acted as no other means known by me have. It has been more successfully used than has any or all other remedies. In my book on etherization in child-birth, p. 307, are ten cases of puerperil convulsions in which anæsthetics were used. Of these, six of the women did well, and three children were born alive. In another table, p. 330, are seven cases of puerperal convulsions, in which etherization was not used. Of these, six women died, and only one child was born alive. Now these are not picked cases. They occurred in the same year as did those in the other table in which etherization by chloroform was used. They are too many to be resolved into coincidences, and my latest observations of puerperal convulsions, furnish new evidence of the safety and importance of the use of chloroform in this disease. The latest trials of it have exceeded in interest the preced[illegible] ones.

For in some of these, chloroform has been employed *at the very beginning* of the attack, and before any other means have been used, as bleeding, &c., and with the happiest results. In some of my latest cases, before I saw the patient, most faithful medication had been tried, and where the case seemed utterly hopeless. Chloroform has been curative. My friend, Dr. Crane, of East Boston, will recollect cases of this kind, apparently utterly hopeless, in which the immediate result of its use was permanent suspension of the fits, and rapid recovery. I am very glad of an opportunity to give this direct and strong statement of the whole good agency of chloroform in this fatal malady; and I believe I may recommend it to others on an amount of testimony on which they may rely, without going into the evidence. I will say, that chloroform in these cases has been far more, and immediately useful than has sulphuric ether. I have seen the two tried, fairly tried, and am sure of what I say. In the convulsions of children, chloroform has also been useful. I have tried the old, the stereotyped plan—bleeding, bathing, vomiting, purging—faithfully tried, and have known the fits steadily to go on. Under advice or consultation this treatment has been continued, and in vain. I have known fully the beneficial effects of chloroform in these cases. I have been informed of cases in which chloroform has been used in the *first instance* in fits of children, and of its good effects in such exclusive use of it. In the progress of a case of diseased brain, in a child three or four years old, and in which effusion was believed to exist, convulsion became a most distressing complication. The mother, advanced in pregnancy—looking daily for labor, asked if some means could not be used to diminish such constant and terrible expressions of suffering. *Sulphuric ether* was tried in this case. The convulsions were lessened in frequency, and soon ceased This child recovered. In the use of such an agent as chloroform in such cases, the greatest care should be observed. Sponge should never be used for applying it. *I never saw sponge used abroad.* A bit of cloth, folded on a handkerchief as taken from the drawer, is always used. Very little is poured, or, better, *dropped* upon its centre, and the cloth is always so held as to allow a due mixture of atmospheric air with the chloroform vapor. As soon as its effects are perceived, the handkerchief is removed. Unless such and all other needed cautions are used, it is not to be wondered at that untoward, even fatal results should be produced.

I have spoken of the use of chloroform in Europe, as I saw it used and heard it spoken of. I have alluded to the general confidence in its safety, and of the questions which now and then arise there concerning this important point in the employment of this active medicinal agent. While writing I have received Rev. Dr. Parker's Report of his Hospital in Canton, China, for 1850 and 1851, and this pamphlet from a distant land has a word concerning chloroform. In 17 surgical operations—9 for stone, and 8 for the removal of tumors, some of enormous size—chloroform was used, producing entire insensibility, and without any untoward occurrence. No such occurrence is alluded to in the pamphlet, which considering its source, is the best evidence that such has not been met with in Canton. I state the grave character of the operations, the length of time required, and but for chloroform, the exquisite suffering. I refer to this especially, because most of the mortality after its use elsewhere has occurred when it has been employed as a *preparation* for the most trifling operations in surgery, when the general health was good, or had not been impaired, nor the nervous impressibility lessened by preceding disease and suffering. I mention this Report, not merely to allude to the important professional facts it contains, but to express my grateful sense of Dr. Parker's kindness in sending it to me. Rev. Dr. Parker is at the head of the Canton Hospital, which in 1850 received 4712 patients, and in 1851, 4103, about one half of which were for diseases of the eye. Dr. Parker treats all cases without fee, whether in or out of the Hospital, and whether poor or rich, and with extraordinary success. He is training native pupils for the same service, and this, too, with encouraging success. In this way he has secured an an interest in his missionary labors which is very striking. The evidence is in the letters of thanks of those he has cured, and which are in this Report. You see how these converts love him, with what deep reverence they allude to the sacred writings which he is unfolding and explaining to them, and you cannot withhold from him your admiration, your interest in the success of his extraordinary labors. The quantity of chloroform used by Dr. Parker, was small, sometimes half a drachm only, at others two or three drachms.

I have stated above what I saw done with chloroform in Edinburgh, and what is recorded of it in Canton, how common its use, and how safe has been its employment in those cities. But it is as well known that death elsewhere has followed its inhalation, in too many cases (between thirty and forty), and too soon after its exhibition to leave any doubt that it was the cause of those deaths. This fact in the history of chloroform has induced many medical men to abandon its use entirely in any of its forms, and has confined its employment by others to few and rare cases.—*Prof. Channing's Professional Reminisences of Foreign Travel.*

